# Regenerative Endodontic Management of an Immature Molar Using Calcium Hydroxide and Triple Antibiotic Paste: a Two-Year Follow-Up

**DOI:** 10.1155/2020/9025847

**Published:** 2020-02-10

**Authors:** Mohannad Alasqah, Sulthan Ibrahim Raja Khan, Khalid Alfouzan, Ahmed Jamleh

**Affiliations:** ^1^King Saud Hospital, Unaizah, Saudi Arabia; ^2^Restorative and Prosthetic Dental Sciences, College of Dentistry, King Saud bin Abdulaziz University for Health Sciences, National Guard Health Affairs, Riyadh, Saudi Arabia; ^3^King Abdullah International Medical Research Centre, National Guard Health Affairs, Riyadh, Saudi Arabia

## Abstract

The regenerative endodontic procedure (REP) is considered a viable treatment option for immature teeth with necrotic pulp and periapical radiolucency which can facilitate continued root formation. In this report, an immature necrotic mandibular molar received REP in three appointments wherein chemomechanical debridement was performed with a sequential application of nonsetting calcium hydroxide (in the whole canal) and triple antibiotics paste (in the root's middle third) dressings in the first and second appointments, respectively. In the third appointment, blood clots were created in the root canals. MTA was placed over the blood clots and the tooth was restored with a composite filling and stainless-steel crown. Recall appointments were performed for two years where the tooth was deemed asymptomatic clinically and a complete root formation with significant periapical healing was evident radiographically. More cases are required to warrant the feasibility of this disinfection protocol.

## 1. Introduction

Pulp necrosis in immature teeth usually results in incomplete root development with open apices and thin walls that make endodontic management challenging [1]. In the past, the apexification procedure with calcium hydroxide (CaOH) was adopted. However, this procedure requires intracanal placement of CaOH for the long term which leads to weakening and brittleness of the dentinal wall [2]. Also, it requires multiple appointments where a patient's compliance and contamination are critical issues. Single-step MTA apexification was introduced as a better treatment option for teeth with open apices as it overcomes the previous drawbacks and is practiced as a more predictable treatment with a higher success rate [3]. However, neither CaOH apexification nor MTA apexification can allow the physiologic root development, resulting in a fragile and thin root [4]. Regenerative endodontic procedure (REP) is another treatment modality for necrotic immature teeth that is designed to allow the root to continue the development [5, 6]. A retrospective study found survival of all teeth treated with REP compared to 77% of teeth treated with CaOH apexification [4].

Several published cases with immature roots [5–9] indicate that the REP has the potential to encourage the continued formation of the root width and length. This procedure involves proper infection control, a suitable matrix for fresh tissue ingrowth, and adequate coronal seal [7]. The proper infection control was reported with different methods of disinfection, such as the use of CaOH or triple antibiotics paste (TAP). A systematic review found that CaOH alone was used in 13% of the included case reports while the TAP accounts for 86% of the reports [10].

This case report describes the REP for management of an immature permanent molar tooth with necrotic pulp, wherein both CaOH and TAP were sequentially used.

## 2. Case Report

**Figure 1:**
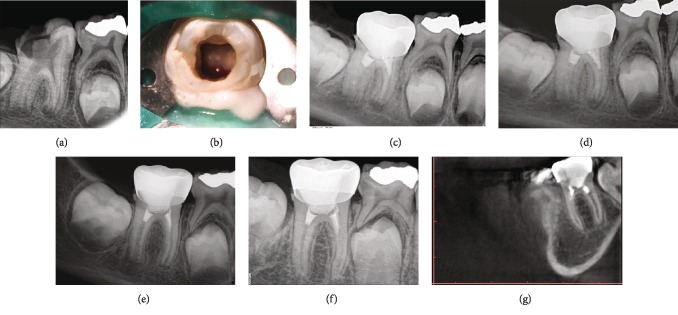
The mandibular right first molar had immature roots with periapical radiolucency (a). During the regenerative endodontic procedure, successful bleeding was made in the distal canal only (b). Postobturation radiograph shows MTA plug in the coronal third and stainless crown (c). Follow-up appointments after 6 months (d), 12 months (e), and 24 months (f) showed continued root formation and significant periapical healing. CBCT confirmed the healing (g).

An 8-year-old medically fit boy was referred to the dental center at Riyadh Elm University Hospital, Riyadh, Saudi Arabia, for management of immature permanent mandibular first molar. Clinical examination revealed extensive caries on the occlusal and distal sides without sensitivity to percussion and palpation. Vitality test with Endo-Frost cold spray (Roeko; Coltene Whaledent, Langenau, Germany) resulted in a negative response. Periodontal probings were within the normal limits (<3 mm around the tooth) and no mobility was seen. Radiographic examination revealed that the tooth had immature roots with periapical radiolucency (Figure 1(a)). A diagnosis of necrotic pulp with asymptomatic apical periodontitis was reached. Among the various treatment options, REP was suggested as the best possible option. Benefits and risks of REP were fully explained to the patient and his mother, and the consent was obtained for the procedure.

In the first visit, an inferior alveolar nerve block with 2% lidocaine and 1 : 100,000 epinephrine was given, a rubber dam isolation was made, and caries removal and proper access cavity were performed. Three canal orifices were allocated and cervical preflaring was done using size #3 Gates Glidden to facilitate canal irrigation. The canals were irrigated by 20 ml of 1.5% sodium hypochlorite (NaOCl) for 10 minutes and then dried by paper points to receive nonsetting CaOH (AH Temp, Dentsply, York, PA) was applied to the full length of the canals and the tooth was temporarily restored with Cavit (3M ESPE, Seefeld, Germany).

The second appointment was scheduled after one week. The tooth was found asymptomatic. The access cavity was reopened and the CaOH was washed out of the canals by using a 20 ml of 1.5% NaOCl. The canals were dried by paper points to receive TAP dressing. The TAP was made from a mixture of three antibiotics (ciprofloxacin, metronidazole, and minocycline) with normal saline until creamy consistency was obtained. In order to reduce the discoloration effect of TAP, a dentin bonding agent was placed on the dentinal walls and cured before the TAP application. The TAP was introduced into the middle third of the root only and the access cavity was temporized with Cavit temporary filling again.

Three weeks later, a third appointment was made. The tooth was asymptomatic. In this appointment, anesthetic infiltration of 3% mepivacaine without vasoconstrictor was administered. The TAP was washed out from all the canals and the canals were thoroughly irrigated with 20 ml of 17% EDTA. Following canals drying, bleeding was initiated by inserting a 25-K file (Dentsply Maillefer, Ballaigues, Switzerland) beyond the apex by 2 mm in each canal. In the distal canal, the bleeding filled the canal adequately (Figure 1(b)). In the mesial canals, there was no adequate bleeding and blood was transferred from the distal canal to both mesial canals using a sterile medical injection 27-gauge syringe as described in a previous study [11]. After the blood clot formation, a fast set PD MTA (Produits Dentaires SA, Vevey, Switzerland) was applied over the blood clots by using a microapical placement (MAP) system (Produits Dentaires SA, Vevey, Switzerland). At the same visit, the tooth was restored with a composite core and stainless steel crown by a pediatric dentist (Figure 1(c)).

Recall appointments were scheduled at three, six, twelve, and twenty-four months for the outcome evaluation (Figures 1(d)–1(f). Twenty-four months of recall showed the success of the case; the patient was comfortable and asymptomatic. Clinically, there was no sign of infection or inflammation. Radiographic and CBCT evaluation (Figure 1(g)) revealed obvious healing of periapical lesion with increased root thickness and length and complete apical closure. It is noteworthy that the tooth vitality was positive to cold test after 24 months.

## 3. Discussion

REP was performed on an immature nonvital permanent molar tooth with periapical radiolucency by using a minor modification in the disinfection protocol. The induced blood was transferred from the distal canal to the mesial canals. Favorable clinical and radiographic outcomes were revealed after the 24-month follow-up.

Previously published case reports have differences in the management protocol which were mainly limited to the disinfection protocol of the root canal system. For example, Nosrat et al. [11] and Sonmez et al. [12] reported cases of necrotic immature molar teeth treated by revascularization using TAP. Cehreli et al. [13] and Chueh et al. [14] used CaOH and found successful outcomes in all teeth. Moreover, da Silva et al. [15] showed positive outcomes with the aid of apical negative pressure to disinfect the canal.

Resolution of signs and symptoms, regaining pulp vitality, radiographic evidence of continued root development, and apical narrowing are important goals of successful REP [5, 6]. Many factors play a role in achieving successful REP, such as the presence of vital stem cells, disinfecting the root canal system, and the creation of blood clots in the canals. There are stem cells present in the apical papilla where they have the collateral circulation that keeps them stay alive even when the pulp tissue necrosis. Based on this, creating bleeding in the periapical tissue is required to allow the stem cells to enter and accumulate inside the disinfected canal space. These autogenous cells along with the blood clot will facilitate both pulp regeneration and continuing root tissue formation [16–18]. Also, another study revealed the survival and differentiation of the apical papilla and Hertwig's epithelial root sheath after endodontic infection, with clinical radiographic and histological evidence after REP [19].

In this case, the American Association of Endodontists (AAE) recommendations [20] were followed with a few modifications which included the application of CaOH medication inside the root canals for one week before the TAP application. The treated case showed successful outcomes. However, another two published case reports showed failed REP when the two medications were used [21, 22]. This might be attributed to many reasons, such as inadequate mechanical instrumentation, absence of blood clot, and improper medication placement in the canal.

In the first visit of this case, the root canals were completely filled with CaOH to the periapical area to neutralize lipopolysaccharide produced by anaerobic bacteria and induce continued root formation [23–25]. It is known that *Porphyromonas endodontalis* is commonly isolated species from the root canal and apical lesions [26] which has the ability to induce bone destruction and proinflammatory cytokine secretion [27]. Furthermore, its lipopolysaccharide has been reported to inhibit bone mineralization and osteoblast differentiation [28]. The CaOH releases calcium and hydroxyl ions which provide an alkaline condition in the periapical area [29]. The CaOH has the ability to promote cell viability in osteoblast precursor cells of mouse and suppress bone destruction by attenuating the virulence of *Porphyromonas endodontalis* thereby inhibiting the destruction of osteoblasts and osteoclasts [30].

In the second visit, the TAP placement was limited to the middle third in all canals to avoid the possible deleterious effect of TAP on human periodontal ligament fibroblasts and stem cells which could potentially lead to failed REP [31].

In the third appointment, the canals were washed away, irrigated with EDTA, and then dried. It is worth mentioning that EDTA was used as an irrigant to encourage the survival and proliferation of the stem cells in the apical papilla and facilitate their attachment to the root canal dentinal wall [32]. Afterward, attempts to induce bleeding were done in the three canals. However, no bleeding took place in the mesial canals. The lack of bleeding from the mesial canals might be attributed to the periapical pathology. Therefore, bleeding was transferred from the distal canal to the mesial canals as described in a previous study [11].

The tooth showed a positive response to cold testing. Similarly, a previous study reported positive responses to cold in an immature premolar tooth with a coronal MTA plug close to the cementoenamel level [33].

CBCT has emerged as a precious tool in endodontics due to its reliability, accuracy, and three-dimensional imaging capabilities. The AAE and American Academy of Oral and Maxillofacial Radiology and The European Society of Endodontology Position Statement stated that CBCT should be considered as an adjunct in certain situations such as investigation of teeth with the inconclusive interpretation of two-dimensional radiographs [34]. In this case, the CBCT was taken to investigate the apical tissues. The CBCT confirmed significant periapical healing and complete roots formation.

The coronal seal is an important part of any endodontic procedure to prevent infection of the canals. Due to its biocompatibility and excellent sealing ability, MTA was used above the blood clots close to the cementoenamel junction [7]. The fast-set type was used which enabled us to perform immediate coronal restoration with favorable bonding procedures [35].

Since the tooth was extensively damaged by caries, it was restored with composite filling as core and stainless-steel crown that will ensure the occlusal stability and protection until the adolescence. At that time, a plan for full-coverage restoration will be considered since the MTA was used as a cervical barrier which influences tooth discoloration [36–38].

## 4. Conclusion

A multirooted tooth with necrotic pulps and periapical radiolucency was successfully managed with REP as evident clinically and radiographically, where nonsetting CaOH and TAP were sequentially used to warrant complete disinfection of the canals. Randomized clinical trials are required to warrant the feasibility of this disinfection protocol.
